# Autonomic echoes of student life: a mixed-method inquiry with meta-inferential integration of autonomic function, lifestyle narratives, and health behaviours among medical students

**DOI:** 10.2478/aiht-2026-77-4057

**Published:** 2026-03-30

**Authors:** Dharani Bhaskaran, Suba Anagppan, Logeshwari Vijayagopal, Abeetha Subramanian, Chitra Mourali, Daniel Dev Merlin

**Affiliations:** Dr MGR Educational and Research Institute, ACS Medical College and Hospital, Department of Physiology, Chennai, India; Melmaruvathur Adhiparashakthi Institute of Medical Sciences, Department of Physiology, Melmaruvathur, India

**Keywords:** academic stress, autonomic balance, diet, heart rate variability, medical education, physiological health, sleep, akademski stres, medicinsko obrazovanje, prehrana, fiziološko zdravlje, ravnoteža autonomnoga živčanog sustava, san, varijabilnost srčane frekvencije

## Abstract

Medical students face intense academic and lifestyle transitions that can affect their physiological health and well-being. This mixed-method study explores the interplay between the autonomic nervous system activity, measured via heart rate variability (HRV) and lifestyle behaviours, measured with a 20-item questionnaire (LBQ-20) in 90 first-year medical students in South India. We also gathered narrative responses to understand subjective health perceptions and stress adaptation. HRV parameters, calculated individually for each participant and summarised using group-level means, indicate moderate overall autonomic variability (SDNN of 42.76±18.20 ms and RMSSD of 28.37±13.75 ms), while the mean LBQ-20 score of 9.46±3.16 indicates moderate overall adherence to health-promoting behaviours across the cohort. The correlation between the two is weak and non-significant. Thematic analysis of narratives highlight rigid academic routines, stress, poor sleep, and dietary challenges as major barriers to health. This study suggests that medical student well-being is shaped not only by personal lifestyle but also by systemic and psychological stressors.

## Abstract (hr)

Studenti medicine suočavaju se s intenzivnim akademskim izazovima i promjenama u načinu života, što može utjecati na njihovo fiziološko zdravlje i opću dobrobit. Ovo mješovito istraživanje ispituje međudjelovanje aktivnosti autonomnoga živčanog sustava, mjerene varijabilnošću srčane frekvencije (HRV), i životnih navika, procijenjenih pomoću upitnika (LBQ-20), u 90 studenata prve godine studija medicine u južnoj Indiji. Također smo prikupili narativne odgovore kako bismo razumjeli subjektivne percepcije o zdravlju i načine prilagodbe stresu. Parametri HRV-a, izračunani pojedinačno za svakog sudionika te sažeti na razini skupine pomoću srednjih vrijednosti, upućuju na umjerenu ukupnu autonomnu varijabilnost (SDNN 42,76±18,20 ms i RMSSD 28,37±13,75 ms), a prosječni rezultat LBQ-20 od 9,46±3,16 pokazuje umjerenu opću razinu pridržavanja zdravstveno poželjnih ponašanja u promatranoj skupini. Korelacija između tih dvaju pokazatelja slaba je i statistički neznačajna. Tematska analiza narativa kao glavne prepreke očuvanju zdravlja ističe krute akademske rasporede, stres, nedostatak sna i prehrambene poteškoće. Ovo istraživanje upućuje na to da dobrobit studenata medicine ne oblikuju samo osobne životne navike nego i sustavni te psihološki stresori.

The transition to medical education is characterised by hectic academic demands, lifestyle reshaping, and psychological challenges that could affect the health and well-being of medical students. As future healthcare providers, their mental and physical health directly impacts their own academic performance and the quality of care they train to provide (1). The intense medical curriculum often involves long study hours, high stress, and disrupted routine healthy routines, all of which may compromise both the autonomic nervous system (ANS) and physiological function maintaining homeostasis and stress resilience (2).

Heart rate variability (HRV), a direct measure of ANS activity, quantifies the fluctuations in time intervals between each heartbeat (3, 4). Low HRV generally implies psychological stress, poor sleep quality, and poor lifestyle behaviours, all of which have been increasingly reported among medical students (5).

However, previous studies fail to integrate this objective measure with students’ experiences and lifestyle behaviours like physical activity, diet, stress management, and sleep hygiene as key overall health determinants (6, 7).

Our study sought to address this issue by combining qualitative thematic insights obtained from lifestyle narratives and assessment of health behaviour with autonomic data from HRV analyses among medical students in an Indian context. We also wanted to see if there would be any correlations between HRV and narrated lifestyle choices and/or self-perceptions of well-being in the hope that such an integrated approach would provide a comprehensive understanding of how academic stress affects health and adaptation to a new environment among medical students.

## PARTICIPANTS AND METHODS

### Study participants

Our study included 90 of the 95 invited first-year undergraduate medical students aged 18–21 years (mean age 18.33±0.66 years; 37 male and 53 female) attending a private medical school in South India. Five students were excluded based on self-reported history of diagnosed psychiatric or chronic medical conditions, current use of medications affecting autonomic function, or incomplete HRV or lifestyle assessment data. All participants were informed of the study’s objective, procedures, and the voluntary, anonymous, and confidential character, as well as the right to withdraw at any time without consequences. All gave their informed written consent prior to the participation, and the study was approved by the Ethics Committee of the ACS Medical College and Hospital, Chennai, India (Approval No. 562/2022/IEC/ACSMCH).

### Research design and data collection

**Figure 1:**
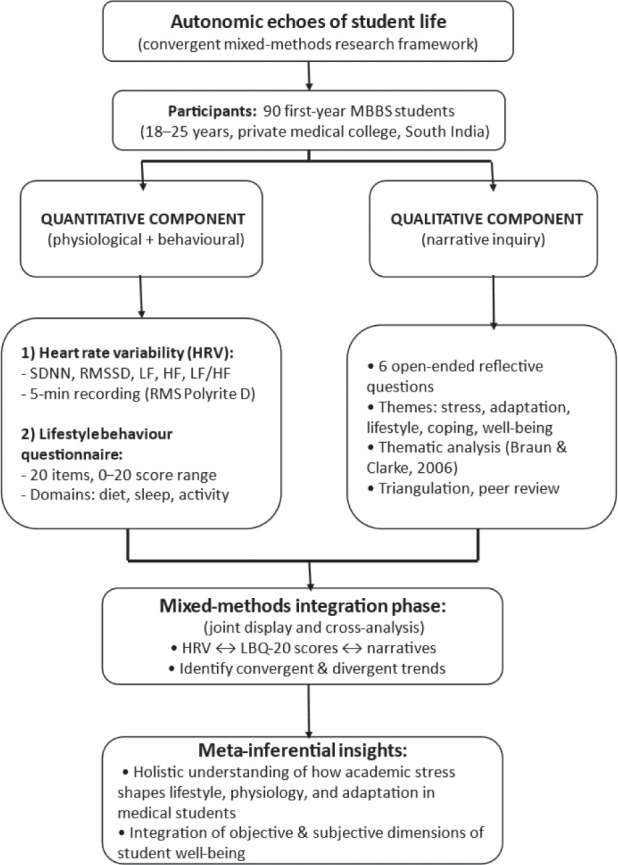
The flowchart of convergent mixed-method study design, illustrating parallel collection of heart rate variability measurements and lifestyle behaviour scoring, and separate qualitative data analysis and final integration

We utilised a convergent mixed-methods design (Figure 1) combining qualitative and quantitative data to reveal complementary dimensions of a shared research problem. The goal was to explore the relationships between ANS activity (quantified via HRV), lifestyle behaviours, and personal narratives. Besides age and gender, we did not collect any other demographic or socioeconomic information.

### Quantitative measures

Following a 10-minute rest in a quiet room maintained at approximately 22–24°C, HRV was measured over 5 min using a Polyrite D instrument and its software (Recorders & Medicare Systems, Panchkula, India) in accordance with the established guidelines for short-term HRV assessment (8). The participants were asked to remain supine and breathe naturally during measurement. The following data were recorded: standard deviation of normal-to-normal intervals (SDNN), root mean square of successive differences (RMSSD), low frequency (LF; 0.04–0.15 Hz), high frequency (HF; 0.15–0.4 Hz), and the fast Fourier transform (FFT) and Lomb-Scargle (Lomb) low-to-high frequency ratio (LF/HF ratio).

In addition, specifically for this study we developed a 20-item Lifestyle Behaviour Questionnaire (LBQ-20) to investigate a broad range of lifestyle behaviours, including physical activity, dietary habits, sleep, preventive health behaviour, stress management, substance abuse (e.g., alcohol consumption and smoking), social connectedness (e.g., continuing supportive relationships), and personal coping and well-being strategies (e.g., responding to stress, opting time for enjoyable activities). Conceptually, it stems from current lifestyle medicine principles, preventive health habits, and WHO guidelines on physical activity, sleep, diet, and stress and draws on existing tools like the FANTASTIC Lifestyle Checklist, Health-Promoting Lifestyle Profile (HPLP-II), and university student health surveys (9,10,11). Also, the final questionnaire was analysed and reviewed by two faculty members in community medicine and physiology to ensure face validity and content relevance, but no formal psychometric validation was undertaken, since it is designed to be used as an exploratory tool rather than a standardised measure.

Each item was scored as either 1, denoting the presence of health-promoting/behaviour, or 0, denoting its absence.

The total score can range from 0 to 20, with higher scores reflecting healthier lifestyle choices. The score of 10 was set as the threshold for dividing the participants in either the low (≤10) or high (>10) score group for further correlation analyses.

### Qualitative data collection

We took the narrative inquiry approach to learn more about the participants’ experiences about health, lifestyle behaviours, and adaptation to change over the first year of study. To this end, we created a study-specific reflective prompt guide. The questionnaire consisted of six open-ended questions: 1) Can you describe your typical day?; 2) What influences your choices regarding diet, exercise, and sleep?; 3) How do you balance academic responsibilities and personal well-being?; 4) In what ways do you think your current lifestyle supports your health?; 5) In what ways do you think your current lifestyle affects your health?; and 6) Have you made any lifestyle changes since joining medical school and if you have, what prompted them?

The responses were thematically analysed following the six-stage process proposed by Braun and Clarke (12) to identify key themes or patterns and integrate (cross-reference) them with quantitative data. This process helped to improve the interpretation of behavioural and autonomic data.

### Mixed-method integration and meta-inferential insights

By integrating quantitative data (LBQ-20 scores and HRV indices) with the results of the thematic analysis we could identify patterns (e.g., low lifestyle scores, low HRV and distress narratives) that called for further correlation analysis.

### Statistical analysis

All analyses were run on SPSS version 26 (IBM Corporation, Armonk, NY, USA). Descriptive statistics were determined for HRV variables and total LBQ-20 scores. The normality of data distribution was checked with the Shapiro-Wilk test and by visually inspecting Q-Q plots and histograms to choose a suitable parametric (Pearson) or non-parametric (Spearman rank) test for further correlation analysis between HRV and LBQ-20 scores.

Statistical significance was set at p<0.05. To ensure a robust interpretation of these differences, we evaluated effect sizes and 95 % confidence intervals.

## RESULTS

Table 1 shows mean HRV power and specific parameters in our sample size. While mean autonomic variability was moderate, the ranges show high individual differences. As software did not provide LF/HF ratios for two participants (P12 and P20), likely due to LF dominance or absent HF signal, we excluded them from this particular HRV parameter.

**Table 1:** Mean heart rate variability by variables in first-year medical students in South India

**HRV variable**	**Mean**	**SD**	**Range**	**N**
Mean heart rate (bpm)	91.73	13.11	63–130	90
SDNN (ms)	42.76	18.2	1.08–118.85	90
RMSSD (ms)	28.37	13.75	5.67–60.44	90
PNN_50_ (%)	13.24	14.87	0–58.9	90
FFT LF/HF ratio	1.85	0.87	0.23–3.96	90
Lomb LF/HF ratio	1.52	1.07	0.45–37.167	88
Total power	590.37	1123.37	8.12–10500.94	90

FFT LF/HF ratio – fast Fourier transform low-to-high frequency ratio; HRV – heart rate variability; Lomb LF/HF ratio – Lomb-Scargle low-to-high frequency ratio; PNN_50_ – percentage of successive normal-to-normal intervals that differ by more than 50 ms; RMSSD – root mean square of successive differences; SDNN – standard deviation of normal-to-normal intervals

**Table 2:** Pearson correlation between LBQ-20 scores and HRV variables in first-year medical students in South India

**HRV variable**	**R**	**p-value**	**N**
SDNN	−0.168	0.113	90
RMSSD	−0.188	0.076	90
FFT LF/HF ratio	−0.093	0.382	90
Lomb LF/HF ratio	−0.042	0.698	88
Total power	0.149	0.163	90

FFT LF/HF ratio – fast Fourier transform low-to-high frequency ratio; HRV – heart rate variability; LBQ-20 – 20-item Lifestyle Behaviour Questionnaire; Lomb LF/HF ratio – Lomb-Scargle low-to-high frequency ratio; RMSSD – root mean square of successive differences; SDNN – standard deviation of normal-to-normal intervals

**Figure 2:**
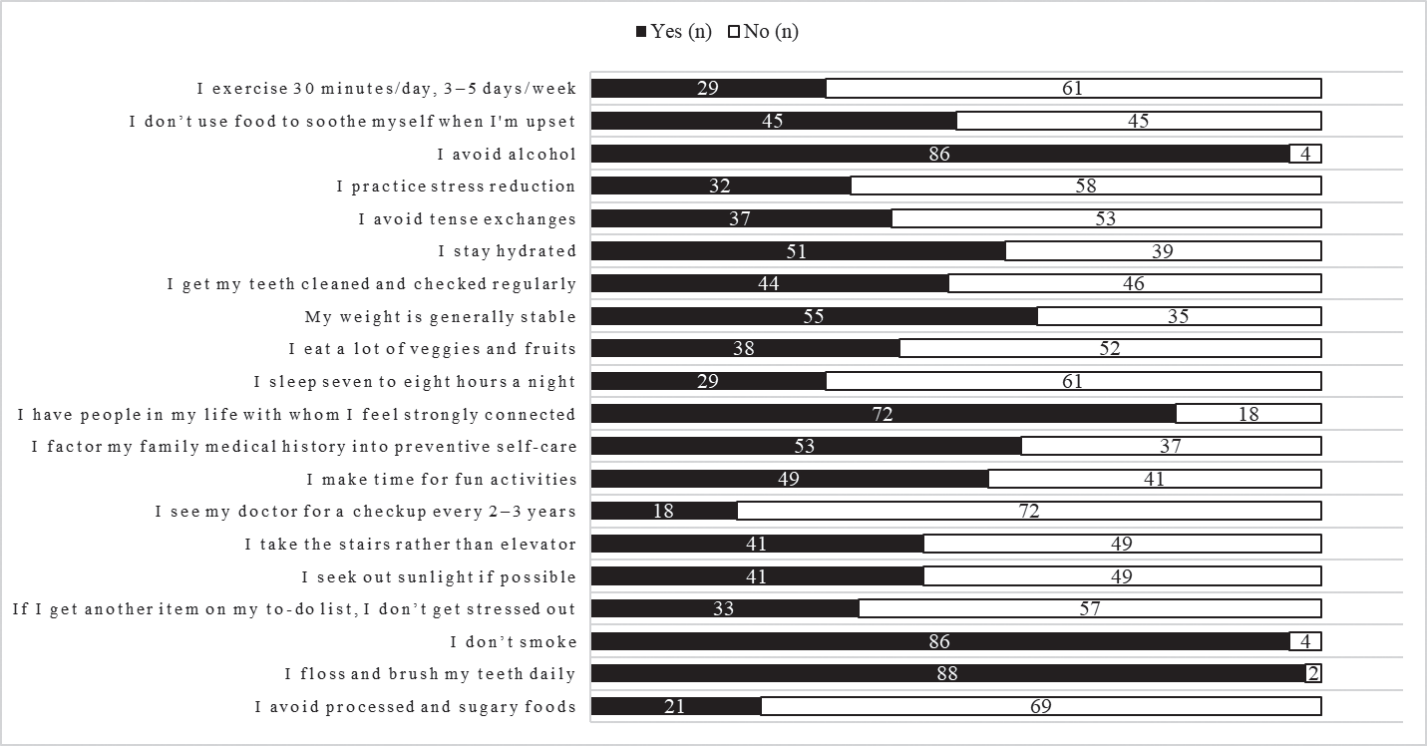
Prevalence of lifestyle behaviours among first-year medical students (N=90)

Figure 2 shows the responses to the LBQ-20 questionnaire on lifestyle behaviour. The mean overall score was 9.46 (SD=3.16; range 1–16; n=90). Highly prevalent adherence to healthy lifestyle choices was reported for daily oral hygiene practices, no alcohol and smoking followed by strong social connections, maintaining stable body weight, and factoring family medical history into self-care. Problematic lifestyle behaviour is revealed by the low self-reported prevalence of regular medical checkups, avoiding sugary and processed foods, adequate sleep, regular exercise, managing stress, and the use of stress reduction techniques.

Pearson correlation analysis revealed no statistically significant correlations between LBQ-20 scores and any HRV metrics. In other words, higher adherence to healthy lifestyle behaviours was not significantly associated with healthy autonomic function.

This prompted us to refine investigation by dividing our participants into low (≤10; n=54) and high (>10; n=36) LBQ-20 score groups to see if the two differed in HRV, but we found no significant differences in any of the metrics (Table 3). Considering that the *t*-test revealed no new information, we believe that future studies should include regression analyses with confounders like gender, age, and socio-economic status. Our negative finding also points to the complexity of autonomic regulation and the possible influence of factors like sleep quality, chronic stress, or genetic variability, which we did not measure.

**Table 3:** HRV Differences in HRV variables between low and high LBQ-20 score groups of first-year medical students in South India (independent *t*-test)

**HRV Metric**	**Low-score group (≤10)**	**High-score group (>10)**	***t*-value**	**p-value**
SDNN (ms)	45.29	39.87	−1.106	0.272
RMSSD (ms)	30.50	26.16	−1.258	0.212
FFT LF/HF Ratio	1.94	1.75	−0.896	0.373
Lomb LF/HF Ratio	1.58	1.44	−0.829	0.410

FFT LF/HF ratio – fast Fourier transform low-to-high frequency ratio; HRV – heart rate variability; LBQ-20 – 20-item Lifestyle Behaviour Questionnaire; Lomb LF/HF ratio – Lomb-Scargle low-to-high frequency ratio; RMSSD – root mean square of successive differences; SDNN – standard deviation of normal-to-normal intervals

### Qualitative findings

Our thematic analysis identified six key themes in participant responses, including 1) highly rigid and academically driven daily routines; 2) influence of stress, energy levels, and social media on lifestyle choices; 3) struggle to balance academic responsibilities and well-being; 4) exercise as a positive influence on health; 5) chronic stress, sleep deprivation, and poor dietary habits as negative influences on health; and finally, 6) changes in lifestyle since the beginning of the medical studies, some for the worse and some for the better (Table 4).

**Table 4:** Narratives on lifestyle and health perceptions among first-year medical students in South India

**Identified themes**	**Summary**	**Example quotes**
Daily routines	Rigid and academia-driven schedules with little time left for self-care, leading to exhaustion	P55: Currently my environment is not very supportive, because I live in a student residence, so sometimes food is not good and sometimes I do not get time for recreational physical activities or sports because of the hectic schedule. P59: A typical day starts around 7:00 a.m. I get ready, have a quick breakfast, and head to the college by 9:00 a.m. Most mornings are filled with lectures or lab sessions until noon. After lunch, I attend more classes. I usually get to the student residence by early evening, take a short break, and then spend a few hours studying or completing assignments. I try to eat dinner around 8:00 p.m. P49: I wake up at 7:00–7:15 a.m., get ready for college and go to college at around 8:45 a.m. I eat lunch at 12:45 p.m. and attend the second half of classes at 1:15 p.m. After college, I go to a gym at 5:00 p.m. and work out for 1 h and 30 min. I return to the student residence at 6:45 p.m. I take a bath and hang around with friends till 8:00 p.m. when I eat dinner. I start studying at 8:15 p.m. and continue till 1:00 p.m., and then I sleep.
Influences on choices	Decisions driven by stress, social media, mood, peers, and hostel diet limitations	P54: Influencers from social media, I like some influencers who follow a structured daily routine. P82: My choices are mostly influenced by my schedule, energy levels, and stress. When I’m busy or tired, I tend to skip exercise, eat whatever’s quick, and sleep later than I should. P84: for food - hunger temptation, exercise - based on health, sleep - based on tiredness and workload
Balancing responsibilities	Students struggle to balance health and academic life: time and stress constraints dominate	P8: Physically and mentally disturbed by the academic stress and stress at home about finances P35: It hinders it by not letting me spend enough time on each aspect where I want to improve.
Lifestyle supports health	Structured routines, exercise, and some social support from friends and family lead to positive habits.	P40: My exercise routine helps me to maintain a good healthy lifestyle P49: My current lifestyle has been both improving my health and destroying my health. Gym is improving my physical and mental health greatly.
Lifestyle hinders health	Academic pressure, sleep deprivation, sedentary lifestyle, junk food, and mental health issues	P36: I consume too much junk food and do very little exercise. P49: Less sleep and more academic stress from medical studies are destroying my physical and mental health.
Lifestyle changes upon enrolment to medical school	Some students adopted healthier lifestyle behaviour, which was difficult to keep due to fatigue/stress	P34: Yes, I joined gym and started eating healthy and reduced weekly consumption of fast food. P52: Tried taking walks for 30 minutes every day, quit six days in because I had more work to do that didn’t allow me these 30 minutes.

### Mixed-method integration findings

Table 5 summarises the interpretation of integrated quantitative and qualitative data. The most notable finding is chronic stress as the dominant factor influencing the autonomic function, possibly by overriding the positive effects of healthy behaviours. This finding is also consistent with the low adherence to managing stress, adequate sleep, and avoiding processed foods.

**Table 5:** Joint display matrix integrating LBQ-20 scores, HRV variables, and qualitative theme analysis among first-year medical students in South India

**Quantitative component**	**Quantitative variable(s): LBQ-20 score / HRV metric**	**Qualitative theme**	**Integration / Interpretation**
LBQ-20 score	Mean=9.46 (SD=3.16): moderate overall adherence to healthy lifestyle practices	Lifestyle supports health	Students report moderate adherence to health behaviours: hygiene and avoidance of smoking & alcohol habits are the most adhered to practices.
LBQ-20 high-score group (>10)	High: Maintaining oral hygiene practices (88), Avoid alcohol (86), Non-smoking (86)	Lifestyle supports health	Preventive lifestyle behaviours were evident in narratives describing gym participation and conscious self-care practices.
LBQ-20 low-score group (≤10)	Processed and sugary food avoidance (21), enough sleep (29), Exercise (29)	Balancing responsibilities	Students struggle to maintain health behaviours due to workload, academic stress, and time constraints.
Comparison between the low and high LBQ-20 score groups	No significant difference in SDNN: (45.29 vs 39.87, respectively; p=0.27)	Lifestyle changes	Despite better lifestyle scores, students show no significant improvement in HRV, indicative of persistent stress or
HRV variable correlations with LBQ-20 score	Weak and statistically non-significant	Lifestyle hinders health	In contrast to reported healthy behaviours, autonomic function remains largely unaffected. This suggests dominant influence of chronic stress and academic strains on autonomic function.
Autonomic imbalance indicator	Lomb LF/HF missing for 2 participants due to absent HF signal	Daily routines	Possibly due to extreme sympathetic activity towed to rigid schedules and inadequate recovery, reflecting physiological strain.
Stress prevalence	Low adherence to stress management (33) and stress reduction practices (32)	Influences on choices	Low adherence to stress management practices, reflecting student narratives of academic burden and fatigue. Stress appears to dominate lifestyle behaviours and might blunt the effect of healthy behaviours, which explains the weak correlation observed with HRV.

LBQ-20 – 20-item Lifestyle Behaviour Questionnaire

## DISCUSSION

Our mixed-method study underscores the complex interplay between lifestyle behaviours, autonomic function, and experiences among first-year medical students, as it reveals that the autonomic balance is not a simple reflection of lifestyle habits but a consequence of contextual stressors rooted in the medical education system, in line with some other reports (13, 14).

The HRV metrics vary across the cohort, with and a trend toward decreased parasympathetic tone, especially when evaluated using RMSSD and PNN_50_ values. Although they do not indicate poor HRV, these values fall slightly below the expected HRV ranges for healthy young adults, suggesting a subclinical autonomic dysregulation (15). However, LH/FH ratios fall within the normal physiological range (~1.85), indicating a slight sympathetic predominance rather than overt dysfunction (16).

Lifestyle scores reveal moderate overall adherence to healthy behaviours, with high adherence in domains like oral hygiene, avoiding alcohol, and non-smoking, and low adherence in key areas like sleep, diet, and physical activity. Yet, the correlation between lifestyle scores and HRV indices is not significant, indicating that lifestyle behaviours alone might not strongly influence autonomic balance in this group.

However, the qualitative data provide a crucial insight into this discrepancy. Student narratives reveal patterns of rigid daily routines, chronic academic stress, emotional burden, and environmental challenges related to student residence living, which collectively act as persistent stressors (17, 18).

**Figure 3:**
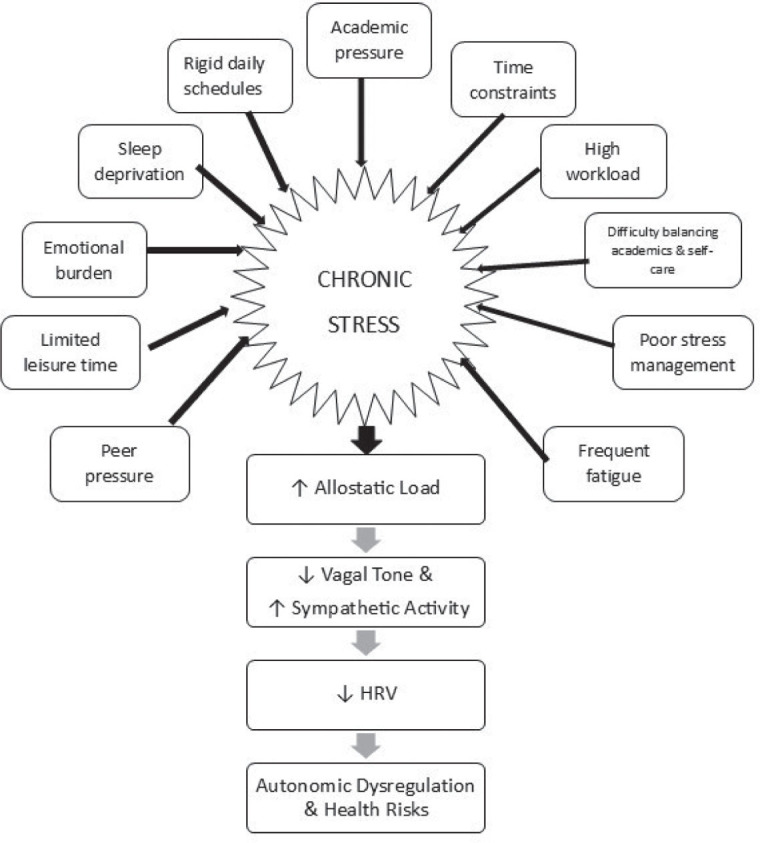
Pathways linking lifestyle disruptions, chronic stress, and autonomic balance based on integrated qualitative and quantitative findings

This phenomenon aligns with existing physiological mechanisms. Continuous exposure to stress activates the hypothalamic-pituitary-adrenal (HPA) axis and the sympathetic nervous system, leading to sustained allostatic load, chronically high catecholamine and cortisol levels, and ultimately autonomic imbalance (low HRV) due to diminished vagal tone (19, 20). Even in students who are engaging in regular health-promoting behaviours, this allostatic pressure might override the physiological benefits of those lifestyle behaviours (21). This is in line with our previous findings suggesting that student well-being is shaped and influenced not only by personal choices but also by environmental and social factors (22). However, our study also suggests that external academic and environmental stressors do not nullify the autonomic benefits of healthy lifestyle behaviours, especially those directed towards stress management, as reported by some other authors (23). Perhaps Figure 3 best illustrates our argument, as it shows the likely way in which chronic stress may impact autonomic function.

Our findings also underscore the need for a shift in medical education, starting with changes in the curriculum that might promote time for rest, wellness-focussed activities, and deeper learning about stress management and sleep. Such strategies are consistent with the current recommendations advocating resiliencecentred reforms in medical education (24, 25).

Recognising and addressing these systemic influences is critical, not only for improving students’ well-being and academic success but also for lowering the risk of early cardiometabolic diseases in future physicians (26).

### Study limitations

We would like to acknowledge several limitations of our study. First, the LBQ-20 questionnaire was created specifically for this study and had not undergone any formal psychometric validation. This may have introduced measurement bias and affected our lifestyle assessment. Second, the small sample size of 90 students from a single institution restricts statistical power and generalisability of our findings. Future studies should therefore include validated lifestyle tools and larger and diverse samples to strengthen their robustness. Third, although the narrative responses reveal plausible causal pathways, the cross-sectional design of this study cannot establish temporal or causal relationships. Fourth, we have not accounted for potential confounders like sleep duration, BMI, gender, and living or social conditions of our students, yet these factors could have affected both HRV and lifestyle behaviours and need to be considered in future studies.

## CONCLUSION

While quantitative data failed to confirm associations between lifestyle scores and HRV metrics, the qualitative narratives exposed critical contextual stressors such as academic pressure, insufficient sleep time, and environmental constraints which likely mediated shifts in autonomic balance. Together, our findings suggest that maintaining a healthy lifestyle alone may not suffice to preserve autonomic health against the challenges of highly stressful medical studies. Instead, reforms targeting stress-related aspects of the medical curriculum and structured wellness approaches are essential to foster resilience and long-term well-being among medical students. In this respect, future longitudinal research could better clarify causal relationships and inform improvements in medical curriculum.
